# Comparison of Phasic Store‐Operated Calcium Entry in Rat Slow‐ and Fast‐Twitch Muscle Fibers

**DOI:** 10.1111/apha.70059

**Published:** 2025-05-19

**Authors:** Elena Lilliu, Rocky Choi, Karlheinz Hilber, Bradley Launikonis, Xaver Koenig

**Affiliations:** ^1^ Center for Physiology and Pharmacology Medical University of Vienna Wien Austria; ^2^ School of Biomedical Sciences, Faculty of Medicine The University of Queensland Brisbane Queensland Australia

**Keywords:** calcium, EC‐coupling, skeletal muscle, slow‐twitch muscle, store‐operated calcium entry

## Abstract

**Aim:**

This study investigates the activation and regulation of phasic store‐operated calcium entry (pSOCE) in fast‐ and slow‐twitch skeletal muscle fibers. Specifically, we aimed to enhance the sensitivity of pSOCE detection in slow‐twitch fibers by optimizing ionic conditions and to compare the physiological relevance of pSOCE between fiber types.

**Methods:**

We employed mechanically skinned fast‐twitch extensor digitorum longus (EDL) muscle fibers loaded with spectrally distinct Ca^2+^‐sensitive dyes to simultaneously measure action potential‐induced sarcoplasmic reticulum Ca^2+^ release and t‐tubular system Ca^2+^ dynamics with millisecond resolution. Experimental conditions were optimized by reducing cytosolic Mg^2+^ and EGTA buffering to enhance Ca^2+^ release in slow‐twitch soleus fibers. Confocal microscopy was used to track t‐tubular system Ca^2+^ depletion and reuptake during electric field stimulation.

**Results:**

Skinned soleus fibers exhibited ~8‐fold lower Ca^2+^ release per action potential compared to EDL fibers, yet pSOCE amplitudes were comparable. Reducing Mg^2+^ and EGTA levels increased Ca^2+^ release and left pSOCE kinetics in EDL fibers unaltered, but enabled pSOCE measurements in soleus fibers. While pSOCE in EDL fibers followed a linear dependence on the ambient Ca^2+^ concentration in the t‐tubular system, such a relationship was violated in soleus fibers.

**Conclusion:**

These findings reveal a novel, fiber‐type‐specific difference in pSOCE regulation. When compared to EDL fibers, soleus fibers exhibited a higher sensitivity to SOCE activation despite releasing less Ca^2+^ from the sarcoplasmic reticulum upon an action potential. These differences may allow soleus fibers to sustain Ca^2+^ homeostasis more effectively, be more resilient against disruptions in Ca^2+^ handling, and entail protection against disease states.

## 1 Introduction

Store‐operated calcium entry (SOCE) is a fundamental mechanism that enables calcium (Ca^2+^) influx across the plasma membrane in response to the depletion of Ca^2+^ stores within the endoplasmic or sarcoplasmic reticulum (ER/SR) [1]. This process is mediated by the stromal interaction molecule 1 (Stim1), which acts as an SR Ca^2+^ sensor [1, 2, 3], triggering the activation of the Ca^2+^‐release activated Ca^2+^ channel, Orai1, on the plasma membrane [2, 4, 5]. While SOCE is well characterized in immune cell activation [1], its precise role in skeletal muscle remains less understood. Notably, SOCE operates relatively slowly in non‐excitable cells [6, 7], whereas in skeletal muscle, it displays much more rapid kinetics [6, 8, 9, 10, 11].

Dysregulation of SOCE results in skeletal muscle pathology. Both loss‐ and gain‐of‐function mutations in Stim1 and Orai1 have been linked to skeletal myopathies [4, 12, 13, 14, 15, 16, 17] and contribute to muscle fatigue [18, 19]. Stim1 and Orai1 are abundantly expressed in skeletal muscle [17, 20] and are known to mediate a chronic form of SOCE (cSOCE) that occurs when SR Ca^2+^ levels are partially or fully depleted. However, complete depletion of SR Ca^2+^ is non‐physiological, as SR Ca^2+^ stores are never entirely emptied during normal muscle function ‐ even following tetanic contractions, and Ca^2+^ levels recover rapidly upon cessation of stimuli [9, 21, 22, 23, 24]. Partial depletion of the SR occurs when the ryanodine receptor (RyR) is leaky, which chronically activates SOCE. RyRs can be leaky due to mutations or post‐translational modifications [25], Nat Comm, accepted [26, 27]. However, the healthy muscle retains a robust SOCE mechanism activated during SR Ca^2+^ release [11] in the absence of a leaky RyR. The physiological activation of SOCE in healthy fast‐twitch muscle was recently demonstrated to be virtually concurrent with SR Ca^2+^ release induced by individual action potentials (APs; [9, 10]). This rapid form of SOCE, which we termed phasic SOCE (pSOCE), exhibits kinetics similar to excitation‐contraction coupling [9, 10, 28].

Fundamental differences exist between skeletal muscle fiber types. Slow‐twitch fibers display reduced voltage‐gated Na^+^ channel density and lower expression of dihydropyridin receptors (DHPRs) and RyRs, leading to less SR Ca^2+^ release and force generation compared to fast‐twitch fibers [29, 30, 31]. Moreover, slow‐twitch fibers also possess a smaller SR and t‐tubular system (t‐system) volume [32] and exhibit different cytosolic and SR Ca^2+^‐buffering capacity [33, 34], which critically affects Ca^2+^ handling dynamics. Importantly, soleus fibers express higher levels of Stim1 as compared to fast‐twitch fibers. Indeed, previous studies using Mn^2+^ quench experiments have reported that SOCE is approximately twice as large in slow‐twitch fibers as in fast‐twitch ones under non‐physiological conditions of a chronically depleted SR [35, 36]. Little is known regarding SOCE across fiber types under physiological conditions, i.e., when SR Ca^2+^ stores are only partially depleted by action potential‐induced Ca^2+^ release, which generates a Ca^2+^ gradient within the SR with its nadir by the Stim1 Ca^2+^‐binding site [24]. Based on the lower SR Ca^2+^ buffering capacity and the purported upregulation of Stim1, we hypothesized a more prominent role of pSOCE in slow‐ compared to fast‐twitch fibers for a given release of SR Ca^2+^. Here, we aimed to establish the experimental conditions required to measure pSOCE in slow‐twitch fibers and to compare its characteristics with those of fast‐twitch fibers.

## 2 Results

In this section, we simultaneously resolve AP‐induced Ca^2+^ release and pSOCE in fast‐ and slow‐twitch muscle fibers with millisecond temporal resolution. This is achieved using mechanically skinned fibers, where the open cytoplasm is exposed to an internal solution containing a Ca^2+^ indicator, while a spectrally distinct Ca^2+^‐sensitive dye is trapped in the sealed t‐system. This setup enables the dynamic tracking of EC coupling and pSOCE interactions. Although EC coupling in mechanically skinned fast‐twitch fibers has been well established [37, 38], we here define the ionic conditions necessary to ensure consistent activation of mechanically skinned slow‐twitch fibers via electric field stimulation (EFS).

### 2.1 pSOCE Measurements Under Reduced Mg^2+^ and Ca^2+^‐Buffering Conditions

Slow‐twitch muscle fibers, such as those predominantly found in the soleus muscle, exhibit significantly lower densities of voltage‐gated Na^+^ channels and reduced expression of DHPRs and RyRs. As a result, these fibers are more difficult to excite [31, 37], SR Ca^2+^ release per AP is markedly reduced [29, 30] and depolarization‐induced force responses are substantially reduced [31].

Given these differences, our previously employed experimental conditions for measuring pSOCE, which relied on high cytosolic EGTA Ca^2+^‐buffering [9, 10, 28], were not well suited for detecting the substantially reduced SR Ca^2+^ release in slow‐twitch fibers. In order to enhance the sensitivity and resolution of our measurements, we therefore adapted our methodology. First, we reduced free cytosolic Mg^2+^ levels from 1 mM, the normal resting Mg^2+^ concentration in muscle [31, 37, 39], to 0.4 mM to facilitate SR Ca^2+^ release and mitigate Mg^2+^‐mediated inhibition of the RyR during EC coupling [40]. This adjustment was relevant, as the soleus muscle has been shown to be particularly sensitive to the lowering of Mg^2+^ [31]. Importantly, the chosen Mg^2+^ levels remained well above the threshold required to induce SR Ca^2+^ release independently of EC coupling [31] with comparable levels of free Ca^2+^ in the SR ([Ca^2+^]_SR_) or t‐tubular (t‐) system ([Ca^2+^]_t‐sys_; Figure S1). Second, to better resolve the expected smaller cytoplasmic Ca^2+^‐transients in soleus muscle, we reduced the cytosolic EGTA concentration ([EGTA]_cyto_) from 10 to 1 mM. This concentration is closer to the estimated physiological Ca^2+^‐buffering capacity of muscle fibers [9], particularly in soleus, where parvalbumin—a major cytosolic Ca^2+^ buffer in fast‐twitch fibers—is absent [30, 41]. At the same time, 1 mM EGTA remained well above the 0.2 mM threshold, below which pSOCE detection is compromised [9].

**FIGURE 1:**
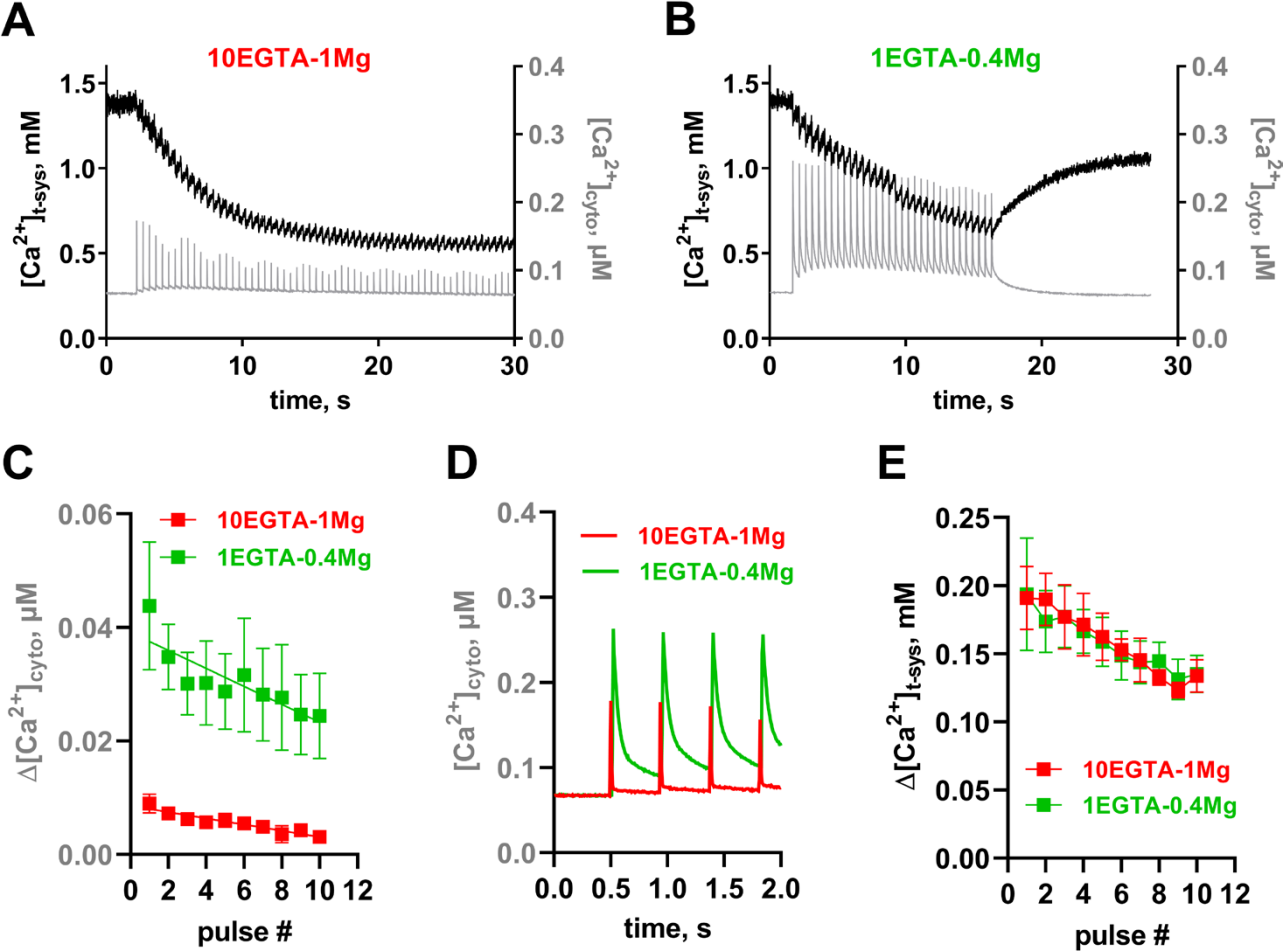
SR Ca^2+^ release and phasic SOCE in skinned rat EDL fibers under reduced Mg^2+^ and Ca^2+^‐buffering conditions. (A, B) Typical recording of [Ca^2+^]_t‐sys_ (left axis) and [Ca^2+^]_cyto_ (right axis) over time as derived from rat skinned EDL fibers during 2 Hz EFS in the presence of either 10 mM EGTA—1 mM Mg^2+^ (A) or in 1 mM EGTA—0.4 mM Mg^2+^ (B). xyt image series of the fluorescent signals of Rhod‐5N and Fluo‐4 trapped in the t‐system and loaded into the cytosol, respectively, were spatially averaged and calibrated (see Methods section). Note that the employed sampling rate was below the Nyquist criterion, which results in an apparent modulation of cytosolic Ca^2+^ transient amplitudes. (C) Change in cytosolic free Ca^2+^ (Δ[Ca^2+^]_cyto_) for subsequent EFS pulses determined from the cytosolic Ca^2+^ level before and immediately after each pulse (also see Figure 2). (D) overlay of the first four cytosolic Ca^2+^ transients for the two recording conditions. (E) pSOCE amplitude values as determined from the step‐wise t‐system Ca^2+^ depletions (Δ[Ca^2+^]_t‐sys_) for subsequent EFS pulses (also see Figure 2). Data are derived from *n* = 5 EDL fibers.

To assess the impact of these modifications, we performed pSOCE measurements in skinned fast‐twitch extensor digitorum longus (EDL) fibers using a previously established protocol ([9, 10], see Methods). Briefly, the Ca^2+^‐sensitive dye Rhod‐5N was loaded into the t‐system of the fibers before skinning, whereas the Ca^2+^ dye Fluo‐4 was added to the cytoplasmic bathing solution to simultaneously measure t‐system and cytoplasmic Ca^2+^ levels during a train of APs. A typical dual‐fluorescence recording of such a preparation using confocal microscopy is shown in Figure 1A,B. Consistent with previous reports [9, 10, 28], EFS triggered APs in the sealed t‐system to induce EC‐coupling and SR Ca^2+^ release (gray trace, right axis). This resulted in a stepwise depletion of t‐system Ca^2+^ ([Ca^2+^]_t‐sys_; black trace, left axis). Between stimuli and after cessation of EFS, [Ca^2+^]_t‐sys_ recovered due to the activity of the Ca^2+^ extrusion proteins PMCA and NCX [26, 42]. Comparing experiments in rat skinned EDL fibers under the previously used experimental conditions (hereby referred to as “10EGTA‐1 Mg”) and the new ones (“1EGTA‐0.4 Mg”), we observed a substantial increase in cytoplasmic Ca^2+^‐transients due to the reduced buffering and decreased Mg^2+^‐mediated RyR inhibition (Figure 1A,B, gray traces; Figure 1C). This effect becomes even more apparent when we compare overlaid Ca^2+^ transients (Figure 1D). Interestingly, despite these changes, t‐system Ca^2+^ depletion (pSOCE) and reuptake remained unchanged (Figure 1E). Likely, the reduction in Mg^2+^ and EGTA imposes opposing effects that counterbalance each other (Figure S2, showing results for all EGTA and Mg^2+^ combinations). Thus, while lowering Mg^2+^ increases SR Ca^2+^ release and hence pSOCE, lowering of EGTA buffering augments cytosolic Ca^2+^ transients, facilitates t‐system Ca^2+^ reuptake via PMCA and NCX, and thereby blunts pSOCE.

### 2.2 Higher Sensitivity of pSOCE Activation in Slow‐Twitch Soleus Fibers

**FIGURE 2:**
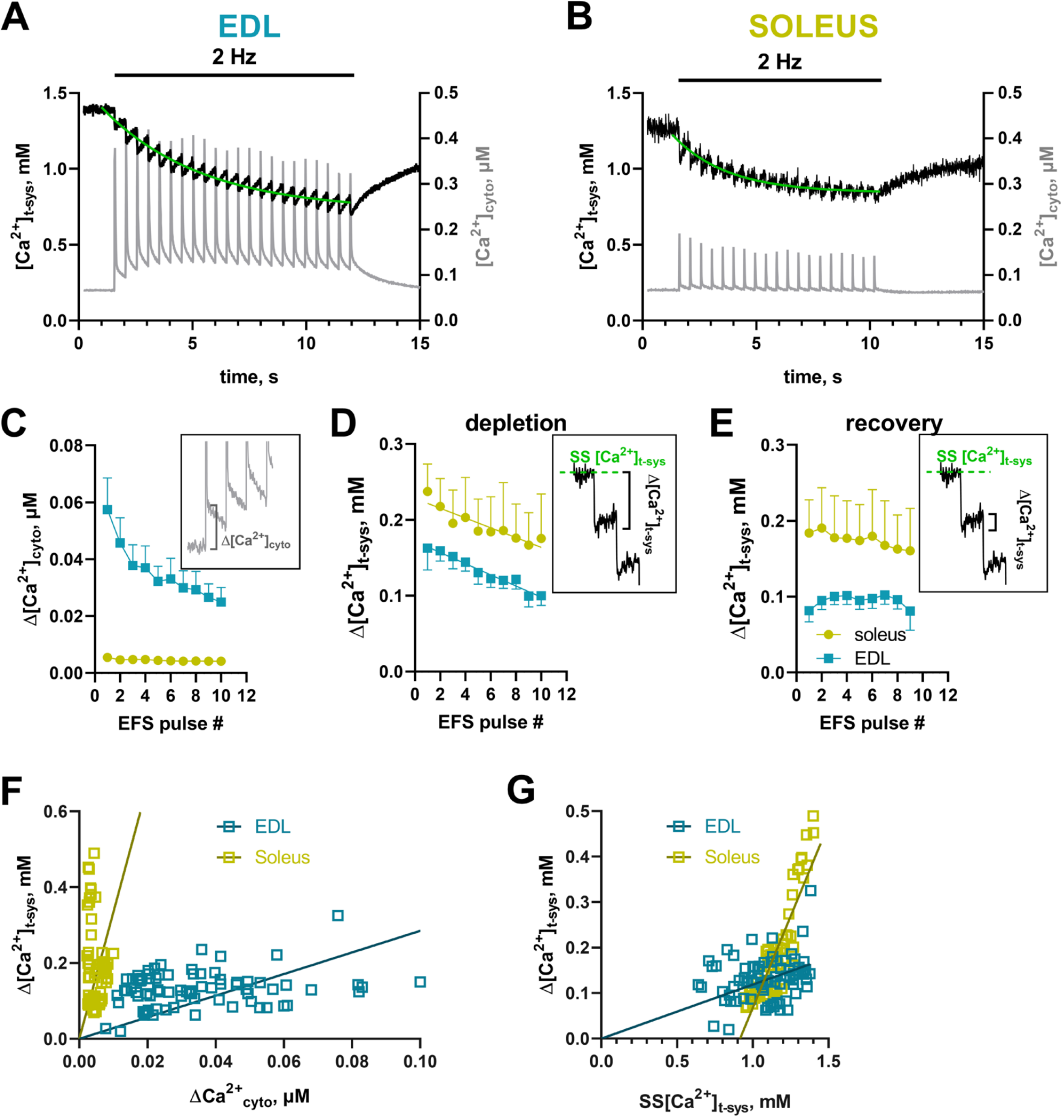
pSOCE in skinned slow‐twitch fibers of rat soleus muscle. (A, B) Typical recordings of [Ca^2+^]_t‐sys_ (left axis) and [Ca^2+^]_cyto_ (right axis) over time as derived from a rat skinned EDL (A) and soleus (B) fibers during 2 Hz EFS under low EGTA and low Mg^2+^ conditions (1 mM EGTA—0.4 mM Mg^2+^). (C) Change in cytosolic free Ca^2+^ (Δ[Ca^2+^]_cyto_) for subsequent EFS pulses determined from the Ca^2+^ level before and immediately after each pulse (see inset). (D) pSOCE amplitude values as determined from the step‐wise t‐system Ca^2+^ depletion (Δ[Ca^2+^]_t‐sys_) for subsequent EFS pulses (see inset). (E) t‐system Ca^2+^ re‐uptake determined as the recovery of [Ca^2+^]_t‐sys_ after pSOCE‐mediated depletion (see inset). (F) Δ[Ca^2+^]_t‐sys_ plotted over Δ[Ca^2+^]_cyto_. (G) Δ[Ca^2+^]_t‐sys_ plotted over the steady‐state Ca^2+^ t‐system levels immediately before depletion (SS[Ca^2+^]_t‐sys_). The slope factor of a linear fit to the data points through the origin represents a relative measure for t‐system permeability (pSOCE mediated), see text for explanation. This dependence fails for the soleus. Data are derived from *n* = 8 EDL and *n* = 6 soleus fibers.

We went on to test our optimized recording conditions on skinned fibers isolated from rat soleus muscle, known to be largely (~85%) composed of slow‐twitch fibers [42]. Using the dual Ca^2+^ dye loading of skinned fibers as described above, we performed measurements of EFS‐induced pSOCE using confocal microscopy. Typical traces of such measurements are shown in Figure 2. For better comparison, we have shown an EDL fiber (Figure 2A) directly next to a soleus fiber (Figure 2B), both recorded under identical recording conditions (1 mM EGTA—0.4 mM Mg^2+^). First, as expected, we noted that EFS‐induced cytosolic Ca^2+^ transients (in gray, right axis) were significantly smaller in soleus compared to EDL fibers. Estimating the difference in SR Ca^2+^ release amounted to an approximate 8‐fold difference (Figure 2C), which is consistent with previous reports (e.g., [31]). Second, we observed the clear presence of pSOCE in soleus fibers, with an amplitude fully comparable to EDL fibers. In fact, when quantifying the individual depletion steps (Figure 2D inset), soleus fibers had slightly larger pSOCE amplitude values compared to EDL despite the much smaller SR Ca^2+^ release (Figure 2D). Importantly, resting [Ca^2+^]_t‐sys_ was not different between soleus and EDL fibers (Figure S3), in agreement with previous reports [42]. Because of a much accelerated Ca^2+^ re‐uptake after each pSOCE depletion (Figure 2E inset), the overall depletion pattern during the whole train of EFS, however, appeared less pronounced (also compare Figure S4 for more original recordings obtained from soleus fibers). To better visualize the difference in pSOCE when comparing EDL and soleus fibers, we plotted the pSOCE amplitudes (Δ[Ca^2+^]_t‐sys_) over the amount of Ca^2+^ that was released from the SR (Δ[Ca^2+^]_cyto_) during subsequent stimuli (Figure 2F). Clearly, comparable pSOCE amplitude values, i.e., t‐system Ca^2+^‐depletions, were observed in soleus muscle fibers at much lower SR Ca^2+^ release values than in EDL fibers.

Finally, we wanted to account for the reduction in driving force that occurs due to the lowering of [Ca^2+^]_t‐sys_ during the train of EFS. In good approximation, the electrochemical driving force is proportional to [Ca^2+^]_t‐sys_. This follows from the Nernst‐Planck equation of electrodiffusion under the assumption of a constant electric field across the plasma membrane as described by the Goldman‐Hodgkin‐Katz flux equation [43]. Here, the Ca^2+^ flux (pSOCE amplitude per unit time) is calculated as

$${\mathrm{Ca}}^{2+}\;\text{flux}\;\left(\text{pSOCE amplitude}\;\mathrm{per}\;\text{unit time}\right)=P_{\mathrm{Ca}}\;\left({\left[{\mathrm{Ca}}^{2+}\right]}_{t-\mathrm{sys}}\;\left(-\upsilon /\left(1-\exp\;(\;\upsilon )\right)\right)-{\left[{\mathrm{Ca}}^{2+}\right]}_{\text{cleft}}\;\upsilon /\left(1-\exp\;(-\upsilon )\right)\right),$$

where P_Ca_ is the permeability of the t‐system for Ca^2+^ and *υ* = 2FV_m_/RT, with *V*_*m*_ the membrane potential and the usual thermodynamic constants. The linear dependence then follows from the assumptions that (i) the resting membrane potential of the fibers is sufficiently negative (the observed excitability of the EDL and soleus fibers implies that the resting membrane potential in our preparation must be comparable to the potential in intact fibers, i.e., around −90 mV) and (ii) that [Ca^2+^]_cyto_, even in the junctional space of the triads, is substantially smaller than [Ca^2+^]_t‐sys_. The above equation then simplifies to Ca^2+^ flux ~ P_Ca_[Ca^2+^]_t‐sys_ [44]. Thus, by plotting Δ[Ca^2+^]_t‐sys_, which is proportional to the Ca^2+^ flux, over the ambient [Ca^2+^]_t‐sys_ (Figure 2G) and determining the slope of the respective relationship, we derived a relative measure of the t‐system Ca^2+^‐permeability underlying pSOCE. While the behavior of EDL fibers is well described by a straight line through the origin, soleus fibers deviated substantially from this behavior and surprisingly intersected far from zero. This behavior was seen in the mean data of all fibers (Figure 2G) as well as when analyzing the individual fibers (Figure S5). These data demonstrate that a different Ca^2+^ permeability underlies pSOCE in soleus and EDL fibers and further suggest substantial differences in pSOCE activation.

## 3 Discussion

Obtaining reliable electrical excitation of skinned soleus fibers has been challenging in the past, particularly when combined with t‐system dye loading, as applied in this study. Here, we have successfully overcome some of these limitations by demonstrating robust EFS‐induced [Ca^2+^]_cyto_ transients using a recording solution with reduced Mg^2+^ and Ca^2+^ buffering capacity.

### 3.1 Higher Sensitivity of pSOCE Activation in Slow‐Twitch Soleus Fibers—Structural and Molecular Determinants

Slow‐twitch soleus fibers exhibit comparable, if not larger, pSOCE amplitude compared to fast‐twitch EDL fibers, despite releasing substantially less Ca^2+^ from the SR during EC‐coupling (Figure 2). Given that pSOCE is activated by SR Ca^2+^ depletion, this finding might appear paradoxical at first glance. One possible explanation could be that soleus fibers inherently maintain lower SR luminal Ca^2+^ levels, making them more susceptible to depletion‐induced SOCE activation. However, evidence suggests otherwise: total endogenous SR Ca^2+^ content is similar in soleus and EDL fibers [45]. Furthermore, soleus fibers exhibit markedly lower levels of calsequestrin‐1 (CSQ1)—the predominant SR Ca^2+^‐binding protein—compared to EDL fibers [33]. This would imply that free [Ca^2+^]_SR_ in soleus fibers is most certainly not smaller and may actually exceed that present in fast‐twitch EDL fibers. While we have previously assessed free [Ca^2+^]_SR_ in EDL fibers (0.7 mM) [10], such measurements have not been performed in rat soleus fibers to date.

A more plausible explanation lies in the fact that the reduced CSQ1 levels in soleus fibers result in a significantly lower luminal Ca^2+^ buffering capacity, leading to a more pronounced drop in terminal cisternae (TC)—the specialized SR regions where SOCE is thought to be initiated [9, 10, 24]—Ca^2+^ concentration upon RyR‐mediated Ca^2+^ release. CSQ1 levels in EDL fibers (~36 μmol/L fiber volume) are more than three times higher than those in soleus fibers (~10 μmol/L; [33]). Notably, while there is expression of CSQ2 in slow‐twitch muscle that is not present in fast‐twitch muscle, this likely adds only ~20% to the total Ca^2+^‐buffering capacity [33]. Hence, the reduced buffering in soleus fibers will allow SR luminal Ca^2+^ levels to decline more rapidly upon stimulation, increasing the probability of Stim1 activation. In addition, the total SR volume in soleus fibers is only about half that of EDL fibers [32], and the same holds true for the TC. This implies that any given amount of Ca^2+^ release will cause a proportionally greater depletion of luminal Ca^2+^ in soleus fibers compared to EDL fibers, thereby accelerating Stim1 activation and SOCE recruitment. Soleus fibers also express higher levels of Stim1 than EDL fibers [42, 46, 47]. Moreover, Stim1L, a long Stim1 splice variant [48, 49] suspected to form pre‐assembled clusters with Orai1 and to underlie SOCE in muscle [9, 10], was found to be about 2‐fold upregulated in soleus muscle [42]. Given the smaller SR volume in soleus fibers, the density of Stim1 per unit of SR membrane could therefore be up to four times higher. While Stim1 is also present in other cellular compartments, e.g., at the nuclear envelop [50], and a detailed analysis of its subcellular distribution in muscle fibers has not yet been conducted, the elevated Stim1 density may facilitate a more rapid and robust activation of pSOCE in response to SR Ca^2+^ depletion in soleus fibers. On the other hand, Orai1 expression does not appear to differ between soleus and EDL fibers [47]. However, similar to the SR, the t‐system volume in soleus fibers is only about half that of EDL fibers [32]. As a result, the density of Orai1 channels per unit membrane area is likely to be significantly higher in soleus fibers, potentially leading to a greater number of pre‐formed Stim1‐Orai1.

Lastly, the smaller t‐system volume in soleus fiber means that even if the absolute amount of Ca^2+^ entering the fiber from the t‐system is twice as low in soleus compared to EDL, it would result in a similar drop in [Ca^2+^]_t‐sys_, assuming comparable Ca^2+^ buffering in the t‐system.

Taken together, these factors—the smaller SR and TC volume, the reduced luminal buffering, the higher Stim1 density, and a potentially increased Stim1‐Orai1 clustering—may help explain why pSOCE activation in soleus fibers is as prominent as in EDL fibers, despite the substantially lower SR Ca^2+^ release per AP.

### 3.2 Physiological Implications and Functional Adaptations

The named molecular and structural differences suggest that soleus fibers rely on a distinct Ca^2+^ handling strategy compared to EDL fibers. Although soleus fibers release less Ca^2+^ from the SR during excitation, their SR Ca^2+^ load is endogenously maintained near maximal levels. We speculate that under these conditions, SERCA function may be strongly reduced due to feedback inhibition [31], and the balance between Ca^2+^ reuptake via SERCA and extrusion via PMCA/NCX may shift toward the latter. Without an effective counterbalancing mechanism, this would lead to significant Ca^2+^ loss from the fiber over time. The increased presence and activity of SOCE in soleus fibers may serve as an important compensatory mechanism to counteract this Ca^2+^ efflux. This idea aligns with reports of higher NCX activity in slow‐twitch fibers [51] and a recent proteomic analysis revealing a 1.4‐fold upregulation of PMCA levels in soleus muscle compared to EDL [46]. Given the smaller t‐system volume in soleus fibers [32], even a modest increase in NCX and/or PMCA activity could have a significant impact on Ca^2+^ extrusion dynamics. This might also explain the accelerated Ca^2+^ reuptake kinetics observed in soleus fibers in Figure 2. Moreover, a similar interplay between enhanced SOCE activity and increased Ca^2+^ extrusion has been documented in muscle fibers with leaky RyRs from malignant hyperthermia patients [26], suggesting that this mechanism may help prevent excessive Ca^2+^ loss under conditions of elevated Ca^2+^ flux to maintain fiber Ca^2+^ content [27].

### 3.3 Dynamic Regulation of SOCE and Future Directions

The relationship between SOCE activation and cytoplasmic Ca^2+^ dynamics in soleus fibers appears to be more complex than a simple linear dependence on the driving force of Ca^2+^ depletion. Theoretical models based on the Goldman‐Hodgkin‐Katz flux equation [43] predict a linear correlation that intersects at zero between t‐system Ca^2+^ depletion (Δ[Ca^2+^]_t‐sys_) and steady‐state t‐system Ca^2+^ levels (SS[Ca^2+^]_t‐sys_), assuming that permeability remains constant. However, in our measurements, this was clearly violated in soleus fibers (Figure 2).

Since SOCE is driven by the unbinding of SR luminal Ca^2+^ from Stim1, the fractional occupancy of Stim1 determines Orai1 channel availability and, thus, overall SOCE‐carried t‐system Ca^2+^ permeability. During AP‐induced SR Ca^2+^ release, Stim1 occupancy is expected to change dynamically. If a steady state is reached, Δ[Ca^2+^]_t‐sys_ should follow a straight line relative to SS[Ca^2+^]_t‐sys_. However, if a steady state is not established, Stim1 occupancy and associated SOCE permeability may change non‐linearly. Our data suggest that in soleus fibers, these changes are more pronounced than in EDL fibers, possibly involving the lower end of the Stim1 occupancy curve. This is reflected in the concave nature of the Δ[Ca^2+^]_t‐sys_ vs. SS[Ca^2+^]_t‐sys_ curve, which appears less steep at lower SS[Ca^2+^]_t‐sys_ levels (Figure S5B). This suggests that soleus fibers operate within a different dynamic range and that SR Ca^2+^ levels may not reach a quasi‐equilibrium during the applied stimulation train. Future studies should aim to directly measure local SR Ca^2+^ concentrations, particularly within the TC, during these processes. One promising approach is the use of genetically encoded Ca^2+^ sensors fused to TC‐specific proteins, such as G‐CatchER, coupled to Junctional SR Protein 1 [24]. Such studies could provide deeper insights into the regulation of SOCE during EC‐coupling and clarify how soleus fibers fine‐tune SOCE activation to meet their specific functional demands.

### 3.4 Study Limitations

We acknowledge that many of the functional insights and observations regarding the expression of proteins involved in excitation–contraction (EC) coupling, Ca^2+^ handling, and SOCE are primarily based on mouse muscle. While the arguments derived from these models are likely relevant, they may not necessarily apply to rat muscle as used in this study. Another limitation concerns the recording temperature of 22°C, which is considerably lower than physiological body temperature. At higher physiological temperatures, the kinetics of Ca^2+^ handling, including pSOCE, are expected to accelerate. Indeed, Ca^2+^‐handling processes have been demonstrated to be significantly faster at such elevated temperatures both in slow‐ and fast‐twitch fibers, e.g., [30, 42, 45, 52]. Notably, the amplitude of SR Ca^2+^ release—the primary driver of pSOCE activation [9]—remained largely unchanged [30, 53]. However, since we did not investigate temperature‐dependent effects in this study, we refrain from speculating on how our findings might translate to this domain.

## 4 Conclusion

By establishing precise recording conditions to measure Ca^2+^ handling during EC‐coupling in soleus muscle, we have, for the first time, characterized pSOCE in slow‐twitch fibers. Our findings reveal that pSOCE operates differently in slow‐ and fast‐twitch fibers, with soleus fibers exhibiting a higher sensitivity to SOCE activation despite releasing less Ca^2+^ from the SR. This enhanced sensitivity can be explained by a combination of molecular and structural adaptations, including a smaller SR, TC, and t‐system volume, reduced luminal buffering due to lower CSQ1 levels, and potentially increased Stim1 density and enhanced Orai1 clustering. These adaptations likely allow soleus fibers to sustain Ca^2+^ homeostasis more effectively. Thereby, the resilience of soleus fibers against disruptions in Ca^2+^ handling may be enhanced, which could entail protection against disease states.

## 5 Materials and Methods

This study conforms with the good publishing practice in physiology as outlined by Jensen et al. [54].

### 5.1 Animals

Male Sprague Dawley rats, aged 6–9 months, were housed in light, temperature, humidity, and CO_2_ standard conditions with a 12‐h light–dark cycle (light phase between 07:00 and 19:00) and a 22°C–25°C temperature interval. Food and water were provided ad libitum. Before experiments, all rats were killed via CO_2_‐mediated asphyxiation followed by cervical dislocation.

### 5.2 Muscle Fiber Skinning and Phasic SOCE Measurements

After animal sacrifice, the EDL and/or soleus muscle was rapidly harvested, transferred to a glass Petri dish covered with a layer of Sylgard (Dow Europe), and submerged in paraffin oil for individual fiber isolation. This procedure was performed as previously described [9, 10, 42]. Briefly, bundles of fibers were mechanically isolated from the muscle, incubated with a standard Ringer's solution containing 2.5 mM of the low‐affinity dye Rhod‐5N (Thermo Fisher Sc, R14207) for 15 min, and then further dissected into single fibers. Individual muscle fibers were “skinned” by mechanically peeling off the sarcolemma with fine forceps. The skinned fibers were then cut and tied at the ends with knots of 10/0 nylon sutures to allow efficient handling of the fibers. The skinned fibers had an average length ranging from 3 mm to 1 cm. The fibers were then transferred to a custom‐made experimental chamber constructed on top of a 1.5 mm coverslip filled with an internal solution (see Table 1 for composition). The internal solution also contained the high‐affinity Ca^2+^ dye Fluo‐4 (Thermo Fisher Sc, F14200). The fibers were fixed to the chamber by holding the two suture threads down using a pair of insect pins (Fine Science Tools) glued to the surface of the experimental chamber. The fibers were then mounted onto the stage of a confocal microscope (Nikon Eclipse Ti‐2) for further Ca^2+^ imaging (Nikon NIS Acquisition Software). Phasic SOCE measurements were performed from spatially averaged ROIs in xyt‐image series obtained at a scanning speed of 8–8.5 ms/frame. APs were generated using electrical field stimulation (EFS) through a pair of platinum electrodes (each with a 1 mm diameter; 1 cm apart) during fluorescence recording. Rectangular voltage pulses, generated by a GRASS S48 square pulse stimulator, were delivered with a duration of 4 ms, an amplitude of 60 V, and a frequency of 2 Hz in all measurements. Sequential scanning of Rhod‐5N and Fluo4 fluorescence was performed simultaneously to the EFS with excitation wavelengths of 561 and 488 nm, respectively. All measurements were performed at room temperature (22°C).

**TABLE 1:** Formulation of experimental solutions.

Solution	Caffeine	Ca^2+^	Mg^2+^	EGTA	Na^+^	K^+^	Creatine phosphate	ATP	HEPES	BTS
Release	30	—	0.01	50	36	126	10	8	90	0.05
10EGTA‐1 Mg	—	0.000067	1	10	36	126	10	8	90	0.05
1EGTA‐04 Mg	—	0.000067	0.4	1	36	126	10	8	90	—
10EGTA‐04 Mg	—	0.000067	0.4	10	36	126	10	8	90	0.05
1EGTA‐1 Mg	—	0.000067	1	1	36	126	10	8	90	0.05
*F*_min_	—	—	1	50	36	126	10	8	90	0.05
*F*_max_	—	5	1	—	145	3	10	8	10	0.05

*Note:* Solutions are based on Cully et al. [42]. All concentrations are in mM. Mg^2+^ was added as MgO, and Ca^2+^ was added as Ca_2_CO_3_. Note that “Ca^2+^” and “Mg^2+^” refer to the free ionic concentrations in solution. Blebbistatin was used as a contraction inhibitor in pSOCE measurements performed in the soleus muscle [42]. pH was adjusted to 7.1 with KOH in all solutions.

### 5.3 Calibration of Free [Ca^2+^]_cyto_ and Free [Ca^2+^]_t‐sys_

The fluorescent signal calibration was performed as described in [9, 10, 28, 42]. Briefly, [Ca^2+^]_t‐sys_ and [Ca^2+^]_cyto_ were derived from the Rhod‐5N and Fluo‐4 fluorescence, respectively, by calibrating the respective fluorescence intensity. Minimum (*F*_min_) and maximum (*F*_max_) dye fluorescence were determined in the presence of maximal (5 mM) and nominally Ca^2+^‐free solutions (see Figure S3), each containing 25 μM of ionomycin (Sigma Aldrich, I9657) and 25 μM of A23187 (Sigma Aldrich, C7522). The spatially averaged real‐time fluorescent signal (*F*) was converted to [Ca^2+^] using the following formula for single wavelength dyes, assuming quasi steady‐state conditions of Ca^2+^‐binding to the fluorophore for every acquired image within the obtained image series [42, 55],

(1) $$\left[{\mathrm{Ca}}^{2+}\right]={K_{d}}^{*}\;\left(F-F_{\min}\right)/\left(F_{\max}-F\right)$$

where *K*_*d*_ indicates the dissociation constant of the dye for Ca^2+^, 1 μM and 0.872 mM for Fluo‐4 and Rhod‐5N, respectively [9, 42]. *F*_min_ value was typically measured at the end of every experiment. *F*_max_ was calculated using the inverse of Formula (1):

(2) $$F_{\max}={K_{d}}^{*}\;\left(F-F_{\min}\right)+F/\left[{\mathrm{Ca}}^{2+}\right]$$

because proper *F*_max_ acquisition requires a higher spatial resolution than the one normally used under our standard experimental conditions, as *F*_max_ solution application to the fiber causes the formation of vacuoles, hardly visible at lower spatial resolution, which need to be excluded for the *F*_max_ recording. Thus, we performed an independent set of experiments to determine the resting [Ca^2+^]_t‐sys_ for all experimental groups and used this value to derive *F*_max_ from Formula (2).

### 5.4 Measurement of Free [Ca^2+^] _SR_

The method used to load the SR of single muscle fibers was modified from that originally described in intact fibers [56]. Single mechanically skinned fibers were mounted in an experimental chamber and bathed in 67 nM [Ca^2+^]_cyto_ internal solution with 10 μM fluo‐5N AM. 10 μM carbonylcyanide p‐trifluoromethoxyphenylhydrazone and 0.05% Pluronic F‐127 detergent were added to decouple mitochondria and to help disperse the AM ester, respectively. Fibers were incubated for 1 h at 30°C. Thereafter, the solution was exchanged to the same internal solution but without fluo‐5N AM. Fibers were then incubated for an additional 1 h at RT to allow for complete hydrolysis of the acetyl moiety. A *K*_*d*_ value of 403 ± 37.75 μM was determined in situ by exposing fibers to a Ca^2+^ ionophore (50 μM ionomycin) in the presence of varying [Ca^2+^]_cyto_ and fitting a Hill equation to the changes in fluo‐5N fluorescence.

### 5.5 Data Analysis

Data is presented as mean ± SEM. Raw fluorescence data were analyzed using custom‐written MATLAB (R2020, The MathWorks Inc.) scripts. Data presentation and fitting, as well as statistical testing, were performed with GraphPad Prism (version 10, GraphPad Software).

## Author Contributions

**Elena Lilliu:** conceptualization, investigation, methodology, validation, visualization, project administration, data curation. **Rocky Choi:** data curation. **Karlheinz Hilber:** writing – review and editing, supervision. **Bradley Launikonis:** supervision, writing – review and editing, conceptualization, validation, methodology. **Xaver Koenig:** funding acquisition, investigation, conceptualization, writing – original draft, methodology, visualization, validation, writing – review and editing, formal analysis, data curation, supervision, resources, project administration.

## Ethics Statement

All animal experiments performed throughout this study complied with the ethical guidelines and the principles of the Declaration of Helsinki and are covered by the license BMBWF 2020‐0.499.046 and 2022‐0.041.862 granted by the Federal Ministry of the Republic of Austria to X.K.

## Conflicts of Interest

The authors declare no conflicts of interest.

## Supporting information


Data S1.

## Data Availability

The data that support the findings of this study are available from the corresponding author upon reasonable request.
