# Supplementary material for: Comparison of Phasic Store‐Operated Calcium Entry in Rat Slow‐ and Fast‐Twitch Muscle Fibers
Source: Acta Physiol (Oxf). 2025 May 19;241(6):e70059. doi: 10.1111/apha.70059 (PMC12087525; doi:10.1111/apha.70059)
Supplement: Supplementary file 1 — Data S1. [file APHA-241-e70059-s001.docx]

# Supplementary material

## Suppl Fig.1


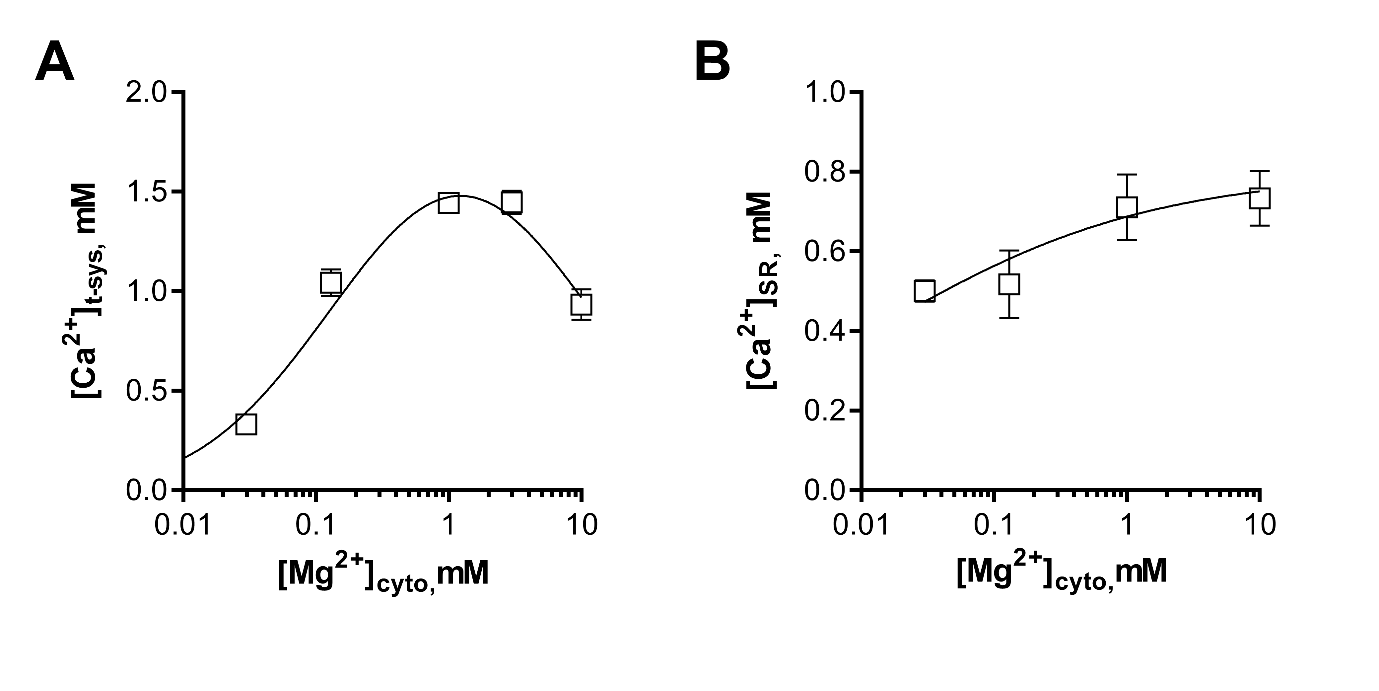


**Suppl Fig.1 | [Ca^2+^]_t-sys_ and [Ca^2+^]_SR_ at different [Mg^2+^]_cyto_.** **A** Mean data of the free Ca^2+^ concentration in the sealed t-tubular system ([Ca^2+^]_t-sys_) at different free Mg^2+^ concentrations in the cytoplasm ([Mg^2+^]_cyto_; 0.03, 0.1, 1,3 and 10 mM) in skinned fibers of rat EDL muscle. Data are derived from the calibrated rhod-5N fluorescence in the t-system. **B** Mean data of the free Ca^2+^ concentration in the SR ([Ca^2+^]_SR_) at different free Mg^2+^ concentrations in the cytoplasm ([Mg^2+^]_cyto_; 0.03, 0.1, 1,3 and 10 mM) in skinned fibers of rat EDL muscle. Data are derived from the calibrated Fluo-5N fluorescence in the SR.

## Suppl Fig.2


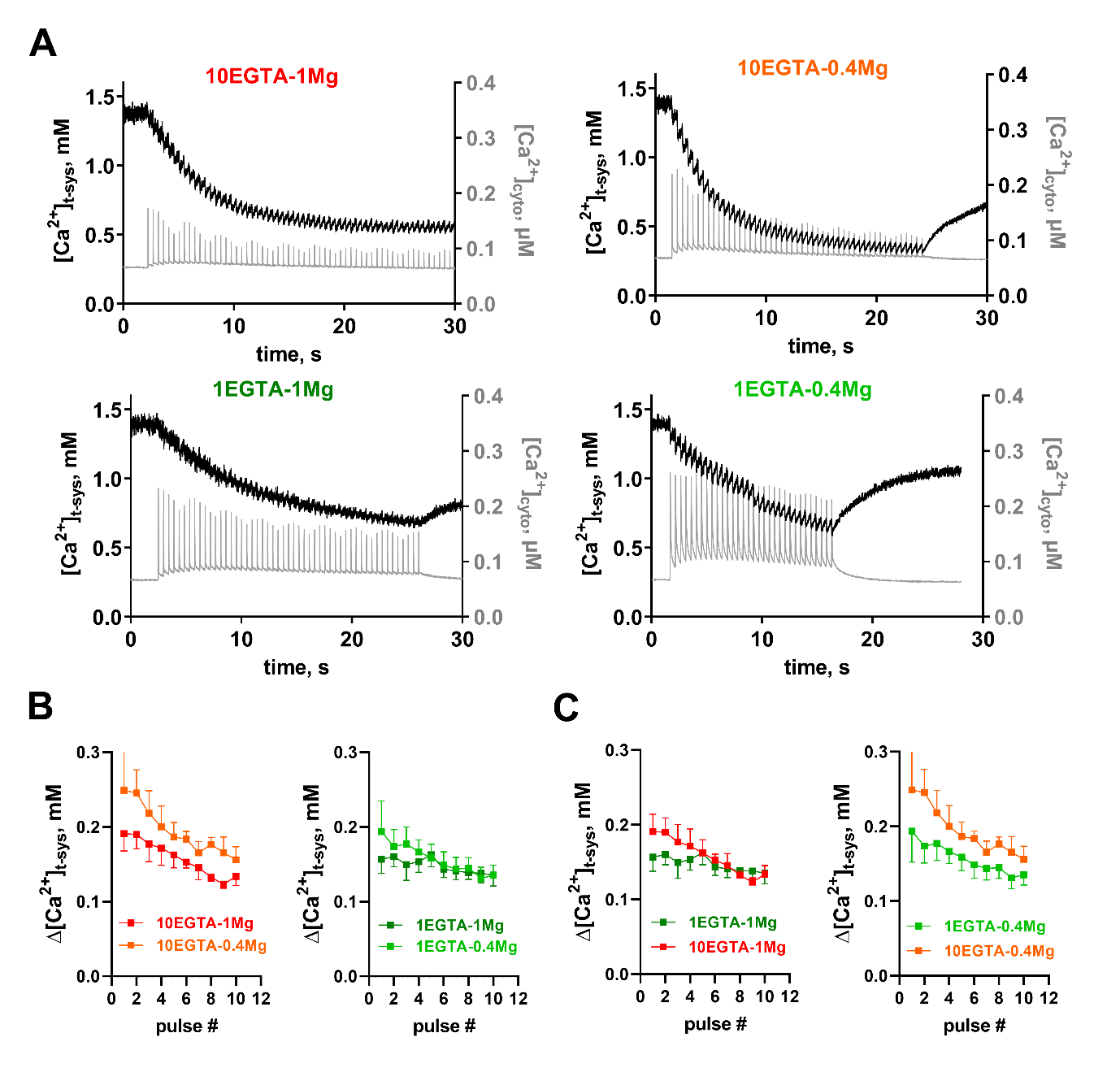


**Suppl Fig.2 | pSOCE measurements in rat EDL fibres using cytoplasmic solutions containing different [EGTA]_cyto_ (1 and 10 mM) and [Mg^2+^]_cyto_ (1 and 0.4 mM).** **A** Original recordings of pSOCE in either 1 or 10 mM EGTA and in either 1 or 0.4 mM free Mg^2+^. **B** Comparison of mean amplitudes of pSOCE (Δ[Ca^2+^]_t-sys_) upon subsequent EFS pulses when reducing Mg^2+^ from 1 to 0.4 mM while keeping EGTA constant at either 10 (left) or 1 mM (right). **C** Comparison of mean amplitudes of pSOCE (Δ[Ca^2+^]_t-sys_ ) during subsequent EFS pulses when reducing EGTA from 10 to 1 mM while keeping Mg^2+^ constant at either 1 (left) or 0.4 mM (right).

## Suppl Fig.3


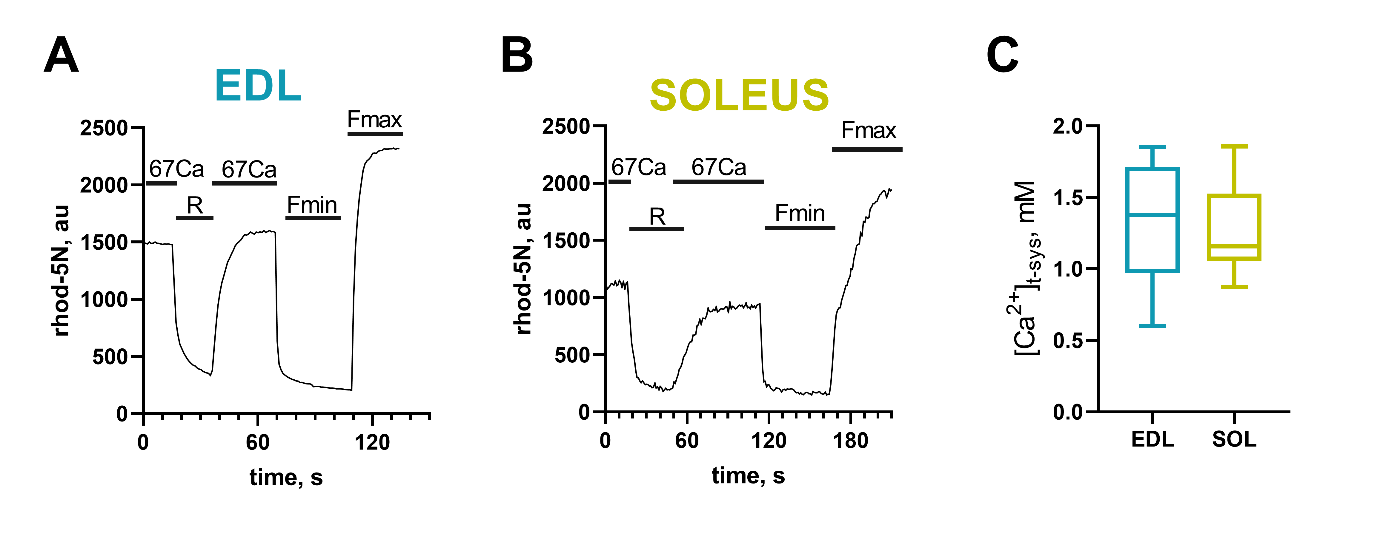


**Suppl Fig.3 | Calibration of t-system rhod-5N in rat skinned EDL and Soleus fibre under low cytoplasmic EGTA and Mg.** Rat skinned fast-twitch EDL (**A**) and slow-twitch soleus (**B**) fibres with t-system-trapped rhod-5N were continuously imaged and exposed to a cycle of a standard solution. Fibres we kept in a low EGTA (1 mM) – low Mg^2+^ (0.4 mM) cytoplasmic solution [Ca^2+^]_cyto_ set to 67 nM (67Ca). A release solution (30mM caffeine, Mg2+-free) was applied before the fibre was reloaded in 67Ca. The fibre was then exposed to the calibrating solutions Fmin and Fmax containing a mixture of ionophores with nominally free and 2 mM Ca, respectively. **C** Box plot of (Ca^2+^]_t-sys_ at 67Ca in EDL and soleus fibres derived from n = 6 and 8 calibrations, respectively.

## Suppl Fig.4


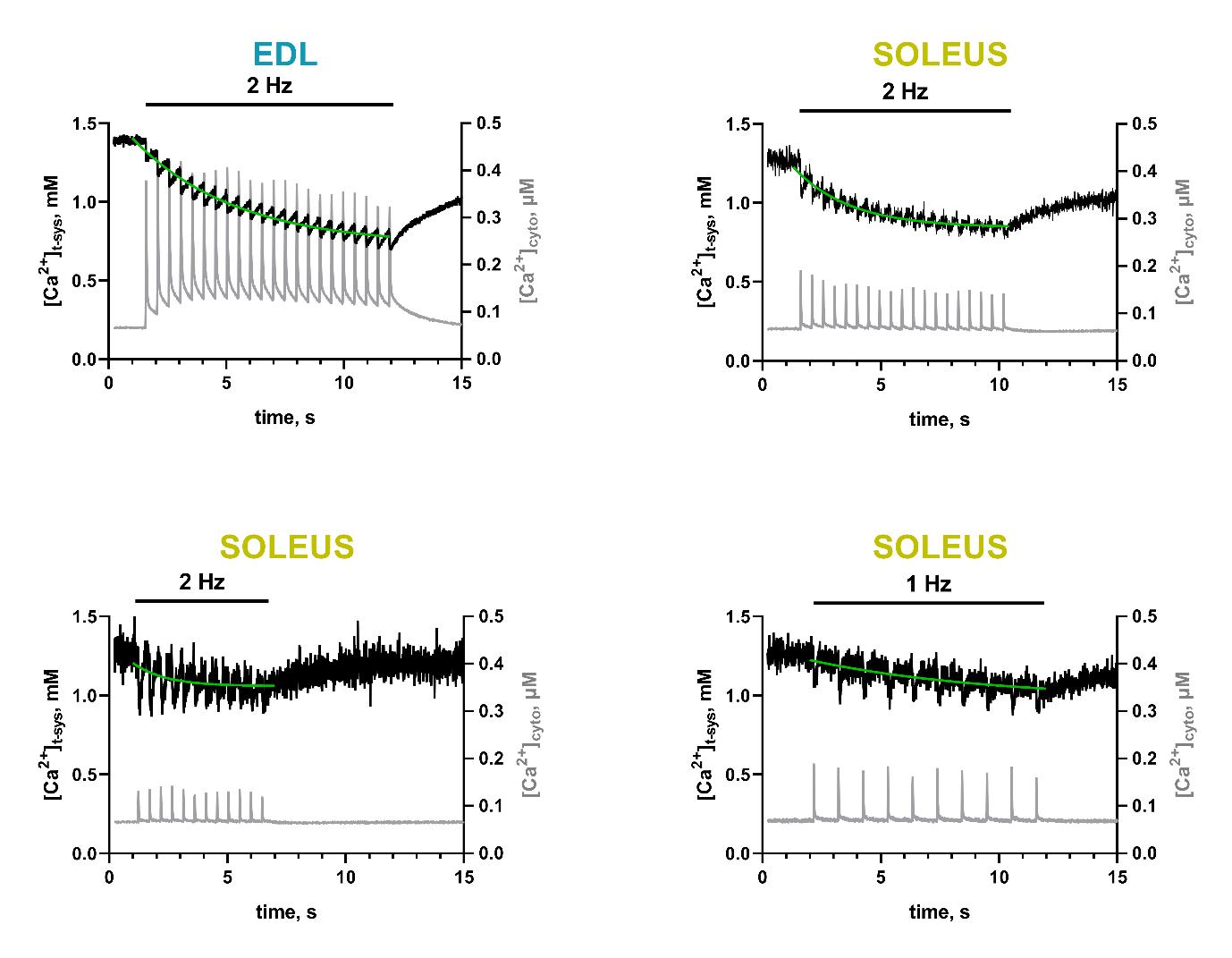


**Suppl Fig.4 | Original recordings of pSOCE in Soleus fibre.** Three recordings of pSOCE elicited with either 1 or 2Hz EFS in three different soleus fibres compared to a recording of pSOCE in an EDL fibre (top left).

## Suppl Fig.5


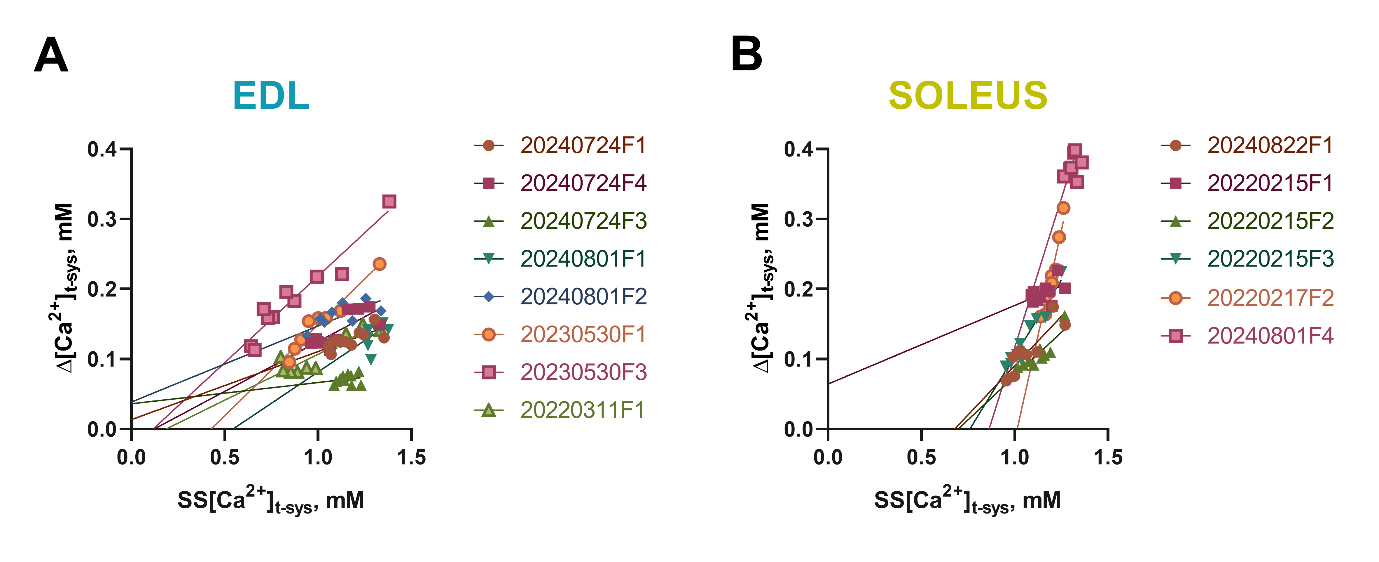


**Suppl Fig.5 | pSOCE underlying t-system Ca^2+^-permeability.** pSOCE amplitudes (Δ[Ca^2+^]_t-sys_) during the first few EFS pulses derived from individual EDL (A) and Soleus (B) fibres. pSOCE amplitudes are plotted over the steady-state Ca^2+^ levels in the t-system (SS[Ca^2+^]_t-sys_) before each depletion to account for the changes in driving force. The pSOCE flux (Δ[Ca^2+^]_t-sys_ per unit time) must follow the Goldman-Hodgkin-Katz flux equation (Hille, 2001) describing electro-diffusion under the assumption of a constant electric field across the plasma membrane. Theoretical considerations predict a linear correlation between pSOCE and steady-state t-system Ca²⁺ levels (SS[Ca²⁺]_t-sys_), assuming that the permeability remains constant. The linear dependence follows from the assumptions that (i) the resting membrane potential of the fibres is sufficiently negative (the observed excitability of the EDL and soleus fibres implies that the resting membrane potential in our preparation must be comparable to the potential in intact fibres, i.e., around -90mV) and (ii) that the Ca^2+^ concentration in the triadic cleft of the cytoplasm is substantially smaller than [Ca^2+^]_t-sys_, which can be reasonably assumed (Launikonis et al., 2009). A linear fit to the data points of individual fibres provides a relative measure of t-system Ca^2+^-permeability underlying pSOCE.

**Table 1: Composition of internal solutions used throughout the study**

| **Solution** | **Caffeine** | **Ca^2+^** | **Mg^2+^** | **EGTA** | **Na^+^** | **K^+^** | **Creatine Phosphate** | **ATP** | **HEPES** | **BTS** |
| --- | --- | --- | --- | --- | --- | --- | --- | --- | --- | --- |
| Release | 30 | - | 0.01 | 50 | 36 | 126 | 10 | 8 | 90 | 0.05 |
| 10EGTA-1Mg | - | 0.000067 | 1 | 10 | 36 | 126 | 10 | 8 | 90 | 0.05 |
| 1EGTA-04Mg | - | 0.000067 | 0.4 | 1 | 36 | 126 | 10 | 8 | 90 | - |
| 10EGTA-04Mg | - | 0.000067 | 0.4 | 10 | 36 | 126 | 10 | 8 | 90 | 0.05 |
| 1EGTA-1Mg | - | 0.000067 | 1 | 1 | 36 | 126 | 10 | 8 | 90 | 0.05 |
| F_min_ | - | - | 1 | 50 | 36 | 126 | 10 | 8 | 90 | 0.05 |
| F_max_ | - | 5 | 1 | - | 145 | 3 | 10 | 8 | 10 | 0.05 |

Table 1 | Formulation of experimental solutions. Solutions are base on Cully et al. (Cully et al., 2016). All concentrations are in mM. Mg^2+^ was added as MgO and Ca^2+^ was added as Ca_2_CO_3_. Note that ”Ca2+” and ”Mg^2+^” refer to the free ionic concentrations in solution. Blebbistatin was used as contraction inhibitor in pSOCE measurements performed in soleus muscle (Cully et al., 2016). pH was adjusted to 7.1 with KOH in all solutions.
